# Clinical Significance of Isolates Known to Be Blood Culture Contaminants in Pediatric Patients

**DOI:** 10.3390/medicina55100696

**Published:** 2019-10-17

**Authors:** Sejong Chun, Cheol-In Kang, Yae-Jean Kim, Nam Yong Lee

**Affiliations:** 1 Department of Laboratory Medicine, Chonnam National University Medical School & Hospital, Gwangju 61469, Korea; sjchun79@chonnam.ac.kr; 2 Department of Laboratory Medicine & Genetics, Samsung Medical Center, Sungkyunkwan University School of Medicine, Seoul 06351, Korea; 3 Division of Infectious Diseases, Department of Internal Medicine, Samsung Medical Center, Sungkyunkwan University School of Medicine, Seoul 06351, Korea; cheolin.kang@samsung.com; 4 Division of Infectious Diseases and Immunodeficiency, Department of Pediatrics, Samsung Medical Center, Sungkyunkwan University School of Medicine, Seoul 06351, Korea; yaejean.kim@samsung.com

**Keywords:** pediatric bacteremia, blood stream infection, contaminant, viridans group streptococcus, Coagulase-negative Staphylococcus, true infection

## Abstract

*Background and objectives:* The objective of this study was to investigate the clinical significance of isolates from blood stream infection known to be blood culture contaminants in pediatric patients. *Materials and Methods:* Microbiological reports and medical records of all blood culture tests issued from 2002 to 2012 (*n* = 76,331) were retrospectively reviewed. Evaluation for potential contaminants were done by reviewing medical records of patients with the following isolates: coagulase-negative Staphylococcus, viridans group Streptococcus, *Bacillus, Corynebacterium, Micrococcus, Aerococcus*, and *Proprionibacterium* species. Repeated cultures with same isolates were considered as a single case. Cases were evaluated for their status as a pathogen. *Results:* Coagulase-negative Staphylococcus had clinical significance in 23.8% of all cases. Its rate of being a true pathogen was particularly high in patients with malignancy (43.7%). Viridans group Streptococcus showed clinical significance in 46.2% of all cases. Its rate of being a true pathogen was similar regardless of the underlying morbidity of the patient. The rate of being a true pathogens for remaining isolates was 27.7% for *Bacillus* and 19.0% for *Corynebacterium* species. *Conclusions:* Coagulase-negative Staphylococcus and viridans group Streptococcus isolates showed high probability of being true pathogens in the pediatric population, especially in patients with underlying malignancy.

## 1. Introduction

Blood culture as the standard in diagnosing bacteremia is a frequently issued test in pediatric patients with suspected infection. A positive blood culture can be a definite tool in detecting blood stream infection. However, the identification of true pathogen is not clear in cases where certain species are identified. Clinical Laboratory and Standards Institute guidelines suggest that coagulase negative *Staphylococcus*, viridans group *Streptococcus*, *Bacillus*, *Corynebacterium*, *Micrococcus*, *Aerococcus*, and *Proprionibacterium* species can be used as evidence of contaminants in blood culture results [1]. However, children with potential immunocompromised status are known to have blood stream infection from coagulase negative Staphylococcus [2,3,4]. Korean studies have shown that among Gram-positive organisms, coagulase negative Staphylococcus and viridans group Streptococcus are most frequently isolated from immunocompromised pediatric blood cultures [5,6]. These findings are of interest. Studies on the process of ruling out contaminants among coagulase negative Staphylococcus can be found in the literature [4,7]. These isolates were noted as contaminants in the past [8]. Viridans group Streptococcus is also noted for its role both as a pathogen and a contaminant in blood cultures [9]. The role of such species acting as a pathogen in pediatric blood stream infection requires further investigation. Thus, the objective of this study was to investigate primary causative pathogens of blood stream infection in a tertiary health care center in a Korean pediatric population, with particular interest in an isolate’s status as a true pathogen.

## 2. Materials and Methods

This was a retrospective review of all blood culture results from patients under the age of 20 that were tested from 1 January 2002 to 31 December 2012. Blood culture samples were incubated using a BacT/ALERT 3D instrument (bioMérieux, Marcy l’Étoile, France). All blood culture specimens were incubated for a minimum of five days before a negative result was considered. When blood culture results showed growth of microorganisms, identification and antibiotic susceptibility tests were performed using an automated VITEK-2 system (bioMérieux, Marcy l’Étoile, France) and/or a MicroScan system (Siemens Healthcare Diagnostics, Erlangen, Germany) accompanied by routine bacteriologic methods. Breakpoints and standards for antimicrobial susceptibility followed guidelines by the Clinical Laboratory and Standards Institute (CLSI) [10,11,12,13]. All results with growth of microorganisms were reviewed. Isolates identified as species of coagulase negative *Staphylococcus*, viridans group *Streptococcus*, *Bacillus*, *Corynebacterium*, *Micrococcus*, *Aerococcus,* and *Proprionibacterium* species were considered as contaminants when isolated only in a single sample. Isolated species was evaluated as a potential pathogen when results were concordant with a secondary blood culture set and the subject’s medical record review suggested blood stream infection. Confirmation as a pathogen was done following a modified algorithm first presented by Richter et al. [7] and guidelines provided by CLSI [1]. Clinical manifestations of blood stream infection were required for these species to be included for analysis as pathogenic microorganisms. The attending physician’s medical records after reporting of the microorganism were considered the main attribute of bacteremia. Acknowledgement of the isolate was considered as evidence of pathogenicity when record of this isolate was found in two or more consecutive days. Simultaneous results from other sites with the same isolates were considered indicative of true pathogenicity. Change of antimicrobial agents after antimicrobial susceptibility test results was interpreted as strong evidence of true pathogenicity. Other references referred for true infection were each patient’s clinical history, white blood cell count, neutrophil count, body temperature, and radiographic interpretation.

The presence of a certain microorganism isolated from blood culture was defined as a single case. In the case of multiple isolates of a particular species, only the first isolate during a single clinical admission was considered as a single case. However, if there was more than a one-month interval between two events and the patient showed proof of improvement from the first blood stream infection episode, the latter isolate was considered as an independent case and included for analysis. Polymicrobial infection was defined as isolation of two or more different species simultaneously from a single clinical episode. Infection related death was defined as death without apparent recovery from the infectious episode regardless of the underlying morbidity. For the analysis of antimicrobial susceptibility, intermediate susceptibility to a particular antibiotic was considered as resistant, in concordance with actual clinical practice.

Subjects were categorized according to age groups. Five age groups were used: under 3 months, 4 to 12 months, 1 year to 5 years, 5 years to 10 years, and older than 5 years old. Medical records were reviewed regarding subject’s underlying morbidity. Assessment for immunosuppression (namely preterm neonates, oncologic patients, patients with congenital immunosuppression, and patients with solid organ transplantation) and infection related mortality was done based on medical records. Patients with congenital anomalies who received solid organ transplantation were counted overlapped (Table 1).

All subjects with underlying cancer were cross checked with their registration status with the Korean National Health Insurance Service (KNHIS) as cancer patients. All cases that expired were also cross checked with data from the government organization, Statistics Korea. Comparison of resistance rate to antimicrobial agents was done by Chi-square test, with *p* < 0.05 considered as statistically significant. All statistical analyses were performed using SPSS 21 (IBM Inc., Chicago, IL, USA). The institutional review board of the study institution waived the requirement for informed consent of subject (SMC 2015-10-001, approval date 3 October, 2015) as this research involved no more than minimal risk to subjects. All personal identification data were censored prior to data collection. This study was approved by the Institutional Review Board of the study institution.

## 3. Results

During the eleven-year period, 76,331 blood cultures were issued to 13,519 pediatric patients and 1961 cases were identified to have growth of potential pathogens in blood cultures. A total of 750 cases (counting polymicrobial infection as separate cases) were identified to be coagulase negative Staphylococcus, viridans group Streptococcus, *Bacillus, Corynebacterium, Micrococcus, Aerococcus,* or *Proprionibacterium* species. After assessment regarding subject’s clinical information was done, 25.9% of isolates included in these criteria were concluded to be true pathogens, with viridans group Streptococcus appearing to show the highest rate of being pathogenic (46.2%). Coagulase negative Staphylococcus was the most frequently isolated and its rate of being a true pathogen was 23.8%. *Bacillus* species were also frequently found to be the cause of bacteremia and 27.7% of all cases that yielded *Bacillus* species isolates had clinical significance, with the majority of them being identified as *Bacillus cereus* (11 cases, 73.3% of all clinically significant *Bacillus* species isolates). Four out of 21 *Corynebacterium* species isolates were true pathogens (14.0%). No case of true infection regarding *Micrococcus, Aerococcus,* or *Proprionibacterium* species was observed. The portion of true pathogens was exceptionally large in patients with underlying malignancy, exceeding 40% in all cases of coagulase negative Staphlyococcus (43.7%, *p* < 0.01 compared to all cases of coagulase negative Staphylococcus isolates). Viridans group Streptococcus also showed large portions of true pathogens in patients with underlying malignancies, although the portion of true pathogens was high regardless of underlying disease (Table 2 and Table 3).

Both coagulase negative staphylococcus and viridans group streptococcus also showed relatively high rates of polymicrobial infection (at around 10%, Supplement Tables S1 and S2).

Penicillin resistance rate for clinically relevant viridans group Streptococcus was 85.4%, in contrast with the resistance pattern for all viridans group Streptococcus isolates (40.0%, data from a previous study, *p* < 0.01 compared to the pediatric population) [14]. This trend was also observed for erythromycin and clindamycin resistance. Coagulase negative staphylococci also showed difference in the rate of antimicrobial resistance in clinically significant isolates. The rate of resistance to erythromycin in clinically significant isolates was 84.2%, in contrast with 50.8% in all isolates (*p* < 0.05). The same tendency was found in clindamycin resistance (62.4% vs. 39.0%, *p* < 0.05). Penicillin and oxacillin resistance was high in both groups, although statistical significance was not found.

## 4. Discussion

We found that the clinical significance of so called “contaminants” was apparently high in the pediatric population. Coagulase negative Staphylococcus, viridans group Streptococcus, *Bacillus,* and *Corynebacterium* showed cases of infection based on our retrospective medical record review. The rate of true bacteremia in this category was high in patients with malignancy (Table 2). This might be due to the fact that patients with malignancy are usually hospitalized with indwelling catheters which may be a cause of the high rate of true pathogens among coagulase negative Staphylococci as they are a part of the normal microflora of human skin. Immunocompromised cancer patients are bound to suffer from higher rates of blood stream infection from these species. Although originally known to be of low virulence, coagulase negative Staphylococus might gain virulence factors [15]. The necessity of infection control measures to a certain species in this category, namely *S. epidermidis*, has been proposed [16]. Although frequently considered as contaminants [8,17], reports of coagulase negative Staphylococcus being pathogens have increased in the literature due to increased portion of catheter related bacteremia and bacteremia in patients with invasive prosthesis [7,18,19,20]. There are multiple reports concerning the rate of true pathogens in this group in nosocomial infection [21,22]. Piukovics et al. reported that out of 161 episodes of febrile cases that yielded coagulase negative Staphylococcus in blood cultures, 50 (31.1%) were assessed to be true pathogens in a population with hematologic disease [23]. Other studies have reported that the rate of contaminants in coagulase negative *Staphylococcus* is 60% [7] to 80% [20], indicating that the remainder of isolates is of clinical significance. Our study showed that 20% of all coagulase negative Staphylococcus were of clinical importance. They were the second dominantly isolated pathogens among Gram-positive pathogens, causing 10% of all pediatric blood stream infection regardless of age group. In comparison, a domestic study investigating the contamination rate in hospitalized patients done by Min et al. [24] stated that contamination rate increased in younger patients. However, their study did not focus on the true contamination rate of coagulase negative Staphylococcus. On the contrary, they included all isolates of these species as contaminants. The true rate of contamination in coagulase negative Staphylococcus in the pediatric population is still largely unknown. The rate of true pathogens among coagulase negative Staphylococcus in children with malignancy is estimated to be around 40%. Clinical strategy regarding these species should be refined as these species are among the most frequently isolated group in blood culture tests. Our results can accredit for the usefulness of correct isolation among species within this group. A recent study displayed different virulence among species in this group [25]. Also, the site of blood draw, along with the number of positive blood cultures, can be a large factor in the pathogenicity of coagulase negative Staphylococcus [26]. Such data were not included in the present study. It would be informative if future studies cover the relationship of the blood draw site and the rate of true pathogenicity of coagulase negative Staphylococcus.

Viridans group Streptococcus are different from other microorganisms analyzed in this study. They are of much importance as pathogens in cancer patients who are neutropenic [27,28]. They are also frequently isolated in the clinical environment [14]. The method we used assigned viridans group Streptococcus as a pathogen when isolation of the same species was done in two or more blood culture sets. This seems to be observed in other clinical environments [7,29], reflecting the fact that viridans group Streptococcus can cause contamination in blood culture during the blood draw process, although they are part of the normal microflora of the human skin and frequently found in the oral cavity, the upper respiratory tract, the female genital tract, and all regions of the gastrointestinal tract. They can act as pathogens when gastrointestinal or respiratory tract mucosa is significantly disrupted and host defense mechanisms are compromised [30]. Less than half of all cases of viridans group Streptococcus bacteremia were proved to be of clinical significance in our study, comparable to other studies done on populations not limited to pediatric patients. Other studies have reported that 30–60% of viridans group Streptococcus bacteremia are clinically relevant [7,31]. Our results revealed that patients with malignancy showed a higher rate of true pathogen in this category, although statistical significance was not reached compared to the whole group. Cases from patients with congenital anomaly or with complications from preterm delivery were too small to assess. However, they did show lower rates of true pathogens. This might be due to the fact that a part of this population is yet to be colonized. This might also be due to the possibility that medical procedures done on this subpopulation did not involve methods that would disturb the integrity of the gastrointestinal tract in these patients. Another factor was that as a large portion of this population had small birthweights and failed to show significant symptoms. Some infection might have been evaluated as contaminants. On the other hand, patients with congenital anomaly and malignancy both exceeded the average rate of being a pathogen. Overall rate of true pathogenicity of viridans group Streptococcus at 46.2% was lower than initially speculated. This might be due to the fact that a large portion of subjects were underweight. In addition, only one set of blood cultures were obtained. We suspect that a portion of these cases might have been dismissed as contaminants.

Members of the Bacillus group were also of note in this study. Cases of Bacillus species related bacteremia have been reported in invasive infections, including bacteremia [32]. This might be due to the underlying morbidity of this study’s population.

Based on these results, we can conclude that the underlying condition of a patient should be considered when assessing the true pathogenicity of an isolate. This would be especially true for pediatric patients with underling malignancies. The rate of coagulative negative Staphylcoccus true infection rate was significantly high. Viridans group Streptococcus should always be treated with due vigilance. The same could be said for other species in this group. Also, the higher rate of antimicriobial resistance in clinically relevant isolates suggests that these isolates might have higher virulence. Further investigation would be required to make a concrete conclusion on this matter.

Limitation of this observation was that it was conducted in a single tertiary hospital with large numbers of neonates admitted for preterm birth and large numbers of cancer patients in children. However, it did show the landscape of serious blood stream infection among isolates frequently considered clinically insignificant, especially if the patient had a condition to be considered immunocompromised. Both coagulase negative Staphylococcus and viridans group Streptococcus were noted as potential pathogens in immunocompromised patients. Along with measures focusing on decreasing the rate of blood culture contaminants, patient’s clinical features are also decisive factors when interpreting blood culture results with isolates of apparently low contamination rate.

## 5. Conclusions

This study describes the potential pathogenicity of various isolates that are often considered as contaminants. Results showed that coagulase negative Staphylococcus and viridans group Streptococcus isolates showed high probability of having true pathogenicity in the pediatric population, particularly in patients with underlying malignancy.

## Figures and Tables

**Table 1:** Patient Characteristics of Positive Blood Culture Results.

Age Group (*n*)	Male (%)	Preterm	Malignancy	Organ Transplantation	Other Immunodeficiency	Congenital Heart Disease	Other Congenital Anomaly
type 0 to 3 months (302)	51.9%	231	9	0	1	75	16
4 to 12 months (152)	50.8%	19	40	25	0	35	31
13 to 60 months (244)	41.9%	6	164	24	3	10	21
61 months to 120 months (203)	67.7%	–	159	2	2	4	4
over 121 months (414)	61.8%	–	318	3	1	2	1
Total (1315)	54.2%	256 (19.4%)	690 (52.5%)	54 (4.1%)	7 (0.5%)	126 (9.6%)	73 (5.5%)

**Table 2:** Assessment for Potential Pathogens.

Microorganism	No.	True Pathogen (%)	Polymicrobial Infection within True Pathogen (%)	Infection Related Death (%)
Coagulase negative Staphylococcus	560	133 (23.8%)	8 (6.0%)	12 (2.1%)
Viridans group Streptococcus	93	43 (46.2%) **	3 (7.0%)	1 (1.1%)
Bacillus species	54	15 (27.7%)	1 (5.9%)	2 (3.7%)
Corynebacterium species	21	4 (19.0%)	0 (0.0%)	0 (0.0%)
Micrococcus species	14	0 (0.0%)	0 (0.0%)	0 (0.0%)
*Propionibacterium acnes*	8	0 (0.0%)	0 (0.0%)	0 (0.0%)
Total	750	195 (26.0%)	12 (1.6%)	15 (2.0%)

Comparison of a certain status within a species/group was done against the total number. Asterisks indicate the level of statistical significance: * *p* < 0.05, ** *p* < 0.01, *** *p* < 0.001, **** *p* < 0.0001. Viridans group streptococcus showed higher numbers of true pathogenicity compared to other groups.

**Table 3:** Portion of True Pathogen According to Underlying Morbidity.

Microorganism	Congenital Anomaly	Complications from Preterm Delivery	Malignancy
Coagulase negative Staphylococcus	18/106 (17.0%)	11/55 (20.0%)	59/135 (43.7%) **
Viridans group Streptococcus	4/7 (57.1%)	1/6 (16.7%)	22/47 (46.8%)
Others	0/5 (0.0%)	0/5 (0.0%)	13/40 (32.5%)

Nominator: no. of true pathogens, denominator: no. of total cases, others: Bacillus, Corynebacterium, Micrococcus, and Proprionibacterium species. Asterisks indicate the level of statistical significance: * *p* < 0.05, ** *p* < 0.01, *** *p* < 0.001, **** *p* < 0.0001. Coagulase negative Staphylococcus showed significantly higher rate of true pathogenicity in children with underlying malignancy.

## Supplementary Materials

The following are available online at https://www.mdpi.com/1010-660X/55/10/696/s1, Table S1: Total Number of Isolated Species; Table S2: Major pathogens according to underlying morbidity.

## References

[1] Clinical and Laboratory Standards Institute (CLSI) (2007). Principles and Procedures for Blood Cultures.

[2] Albano E.A., Pizzo P.A. (1988). Infectious complications in childhood acute leukemias. Pediatr. Clin. N. Am..

[3] McCullers J.A., Williams B.F., Wu S., Smeltzer M.P., Williams B.G., Hayden R.T., Howard S.C., Pui C.H., Hughes W.T. (2012). Healthcare-associated infections at a children’s cancer hospital, 1983–2008. J. Pediatr. Infect. Dis. Soc..

[4] Patrick C.C. (1990). Coagulase-negative staphylococci: Pathogens with increasing clinical significance. J. Pediatr..

[5] Chang M.S., Sung K.W., Kim Y.J. (2011). Clinical characteristics of bacteremia in children with cancer. Korean J. Pediatr. Infect. Dis..

[6] Kang J.E., Seok J.Y., Yun K.W., Kang H.J., Choi E.H., Park K.D., Shin H.Y., Lee H.J., Ahn H.S. (2012). Etiological agents in bacteremia of children with hemato-oncologic diseases (2006–2010): A single center study. Korean J Pediatr. Infect. Dis..

[7] Richter S.S., Beekmann S.E., Croco J.L., Diekema D.J., Koontz F.P., Pfaller M.A., Doern G.V. (2002). Minimizing the workup of blood culture contaminants: Implementation and evaluation of a laboratory-based algorithm. J. Clin. Microbiol..

[8] MacGregor R.R., Beaty H.N. (1972). Evaluation of positive blood cultures. Guidelines for early differentiation of contaminated from valid positive cultures. Arch. Intern. Med..

[9] Dwyer R., Ringertz S. (1997). Viridans streptococci in blood cultures. Can we see any patterns of species related to patient category? Review of 229 cases of positive cultures with viridans streptococci. Apmis.

[10] Clinical and Laboratory Standards Institute (CLSI) (2010). Methods for Antimicrobial Dilution and Disk Susceptibility Testing of Infrequently Isolated or Fastidious Bacteria.

[11] Clinical and Laboratory Standards Institute (CLSI) (2012). Performance Standards for Antimicrobial Disk Susceptibility Tests.

[12] Clinical and Laboratory Standards Institute (CLSI) (2012). Methods for Dilution Antimicrobial Susceptibility Tests for Bacteria That Grow Aerobically.

[13] Clinical and Laboratory Standards Institute (CLSI) (2014). Performance Standards for Antimicrobial Susceptibility Testing.

[14] Chun S., Huh H.J., Lee N.Y. (2015). Species-specific difference in antimicrobial susceptibility among viridans group streptococci. Ann. Lab. Med..

[15] Stach C.S., Vu B.G., Schlievert P.M. (2015). Determining the presence of superantigens in coagulase negative staphylococci from humans. PLoS ONE.

[16] Piette A., Verschraegen G. (2009). Role of coagulase-negative staphylococci in human disease. Vet. Microbiol..

[17] Weinstein M.P., Reller L.B., Murphy J.R., Lichtenstein K.A. (1983). The clinical significance of positive blood cultures: A comprehensive analysis of 500 episodes of bacteremia and fungemia in adults. I. Laboratory and epidemiologic observations. Rev. Infect. Dis..

[18] Martin M.A., Pfaller M.A., Wenzel R.P. (1989). Coagulase-negative staphylococcal bacteremia. Mortality and hospital stay. Ann. Intern. Med..

[19] Rupp M.E., Archer G.L. (1994). Coagulase-negative staphylococci: Pathogens associated with medical progress. Clin. Infect. Dis..

[20] Weinstein M.P., Towns M.L., Quartey S.M., Mirrett S., Reimer L.G., Parmigiani G., Reller L.B. (1997). The clinical significance of positive blood cultures in the 1990s: A prospective comprehensive evaluation of the microbiology, epidemiology, and outcome of bacteremia and fungemia in adults. Clin. Infect. Dis..

[21] Longauerova A. (2006). Coagulase negative staphylococci and their participation in pathogenesis of human infections. Bratisl. Lek. Listy.

[22] Von Eiff C., Proctor R.A., Peters G. (2001). Coagulase-negative staphylococci: Pathogens have major role in nosocomial infections. Postgrad. Med..

[23] Piukovics K., Terhes G., Lazar A., Timar F., Borbenyi Z., Urban E. (2015). Evaluation of bloodstream infections during chemotherapy-induced febrile neutropenia in patients with malignant hematological diseases: Single center experience. Eur. J. Microbiol. Immunol..

[24] Min H., Park C.S., Kim D.S., Kim K.H. (2014). Blood culture contamination in hospitalized pediatric patients: A single institution experience. Korean J. Pediatr..

[25] Cui J., Liang Z., Mo Z., Zhang J. (2019). The species distribution, antimicrobial resistance and risk factors for poor outcome of coagulase-negative staphylococci bacteraemia in China. Antimicrob. Resist. Infect. Control.

[26] Peacock S.J., Bowler I.C., Crook D.W. (1995). Positive predictive value of blood cultures growing coagulase-negative staphylococci. Lancet.

[27] Shelburne S.A., Chaftari A.M., Jamal M., Al Wohoush I., Jiang Y., Abughazaleh S., Cairo J., Raad S., Debiane L., Raad I. (2014). Identification and characterization of catheter-related bloodstream infections due to viridans group streptococci in patients with cancer. Am. J. Infect. Control.

[28] Tunkel A.R., Sepkowitz K.A. (2002). Infections caused by viridans streptococci in patients with neutropenia. Clin. Infect. Dis..

[29] Weinstein M.P. (2003). Blood culture contamination: Persisting problems and partial progress. J. Clin. Microbiol..

[30] Doern C.D., Burnham C.A. (2010). It’s not easy being green: The viridans group streptococci, with a focus on pediatric clinical manifestations. J. Clin. Microbiol..

[31] Corredoira J.C., Alonso M.P., Garcia J.F., Casariego E., Coira A., Rodriguez A., Pita J., Louzao C., Pombo B., Varela J. (2005). Clinical characteristics and significance of *Streptococcus salivarius* bacteremia and *Streptococcus bovis* bacteremia: A prospective 16-year study. Eur. J. Clin. Microbiol. Infect. Dis..

[32] Gaur A.H., Patrick C.C., McCullers J.A., Flynn P.M., Pearson T.A., Razzouk B.I., Thompson S.J., Shenep J.L. (2001). *Bacillus cereus* bacteremia and meningitis in immunocompromised children. Clin. Infect. Dis..

