# Supplementary material for: Clinical Significance of Isolates Known to Be Blood Culture Contaminants in Pediatric Patients
Source: Medicina (Kaunas). 2019 Oct 17;55(10):696. doi: 10.3390/medicina55100696 (PMC6843289; doi:10.3390/medicina55100696)
Supplement: Supplementary file 1 [file medicina-55-00696-s001.pdf]

Table S1. Total Number of Isolated Species

Isolated species	n (%)	Polymicrobial infection (%)	Infection related death (%)
Staphylococcus aureus	227 (17.3%)	11 (4.8%)	11 (4.8%)
Escherichia coli	153 (11.6%)	9 (5.9%)	13 (8.5%)
Coagulase negative Staphylococcus	133 (10.1%)	13 (9.8%)	11 (8.3%)
Klebsiella pneumoniae	114 (8.7%)	16 (14.0%)	12 (10.5%)
Pseudomonas aeruginosa	76 (5.8%)	4 (5.3%)	17 (22.4%)
Enterococcus faecium	74 (5.6%)	9 (12.2%)	21 (28.4%)
Enterobacter cloacae	51 (3.9%)	3 (5.9%)	9 (17.6%)
Enterococcus faecalis	48 (3.7%)	11 (22.9%)	0 (0.0%)
Acinetobacter baumannii	44 (3.3%)	4 (9.1%)	19 (43.2%)
Viridans group Streptococcus	43 (3.3%)	4 (9.3%)	2 (4.9%)
Candida albicans	41 (3.1%)	1 (2.4%)	12 (29.3%)
Streptococcus pneumoniae	37 (2.8%)	1 (2.7%)	3 (8.1%)
Serratia marcescens	22 (1.7%)	1 (4.5%)	2 (9.1%)
Candida parapsilosis	22 (1.7%)	0 (0.0%)	3 (13.6%)
Enterobacter aerogenes	21 (1.6%)	2 (9.5%)	1 (4.8%)
Enterococcus gallinarum	20 (1.5%)	5 (25.0%)	2 (10.0%)
Stenotrophomonas maltophilia	19 (1.4%)	1 (5.3%)	5 (26.3%)
Candida species, others	16 (1.2%)	1 (5.9%)	2 (11.8%)
Bacillus species	15 (1.2%)	0 (0.0%)	2 (12.5%)
Burkholderia cepacia	14 (1.1%)	0 (0.0%)	1 (7.1%)
Klebsiella oxytoca	11 (0.8%)	0 (0.0%)	0 (0.0%)
Streptococcus agalactiae	11 (0.8%)	0 (0.0%)	0 (0.0%)
Others	101 (7.7%)	5 (4.5%)	16 (14.3%)
Total	1315	53 (4.0%)	164 (12.5%)

Table S2. Major pathogens according to underlying morbidity

Congenital anomaly				Complications from preterm delivery				Malignancy			
Species	No.	Polymicrobial infection	Mortality	Species	No.	Polymicrobial infection	Mortality	Species	No.	Polymicrobial infection	Mortality
Staphylococcus aureus	28 (23.9%)	3	8	*Staphylococcus aureus*	27 (13.4%)	1	8	*Staphylococcus aureus*	87 (15.6%)	3	28
Coagulase negative Staphylococcus	17 (14.5%)	1	4	*Enterococcus faecalis*****	26 (12.9%)	5	3	*Escherichia coli*	84 (15.0%)	5	36
Klebsiella pneumoniae	12 (10.3%)	3	5	*Klebsiella pneumoniae*	22 (10.9%)	6	6	Coagulase negative Staphylococcus	61 (10.9%)	7	23
*Enterococcus faecalis*****	10 (8.5%)	4	3	*Escherichia coli*	20 (9.9%)	2	4	*Klebsiella pneumoniae*	50 (8.9%)	3	24
Enterococcus faecium	7 (6.0%)	0	1	*Streptococcus agalactiae*	16 (7.9%)	0	3	*Pseudomonas aeruginosa*	41 (7.3%)	1	23
Viridans group Streptococcus	6 (5.1%)	0	0	*Candida albicans****	15 (7.4%)	0	5	*Enterococcus faecium*	39 (7.0%)	4	22
Enterobacter cloacae	6 (5.1%)	0	0	*Enterobacter cloacae*	13 (6.4%)	0	4	Viridans group Streptococcus	22 (3.9%)	0	9
Candida albicans	5 (4.3%)	0	2	Coagulase negative Staphylococcus	13 (6.4%)	1	3	*Enterobacter cloacae*	19 (3.4%)	3	8
Pseudomonas aeruginosa	5 (4.3%)	1	4	*Enterobacter aerogenes*	9 (4.5%)	1	0	*Acinetobacter baumannii*	18 (3.2%)	1	11
Serratia marcescens	4 (3.4%)	1	0	*Serratia marcescens*	9 (4.5%)	2	3	Bacillus species	14 (2.5%)	1	6
Enterobacter aerogenes	4 (3.4%)	0	1	*Candida parapsilosis*	7 (3.5%)	0	3	*Streptococcus pneumoniae*	13 (2.3%)	1	5
Others	20 (17.1%)	0	5	Others	36 (17.8%)	1	10	Others	124 (22.2%)	4	62
Total	117	7	29	Total	202	11	50	Total	559	21	252

Comparison of a certain status within a species/group was done among underlying condition. Asterisks indicate the level of statistical significance;*$p < 0.05$, **$p < 0.01$, ***$p < 0.001$, ****$p < 0.0001$. *Enterococcus faecalis* was isolated significantly higher in patients with congenital abnormalities and infants with complications, compared to patients with malignancy. *Candida albicans* was also isolated higher in infants with complications.
